# Triple-site rTMS for the treatment of chronic tinnitus: a randomized controlled trial

**DOI:** 10.1038/srep22302

**Published:** 2016-03-01

**Authors:** Astrid Lehner, Martin Schecklmann, Mark W. Greenlee, Rainer Rupprecht, Berthold Langguth

**Affiliations:** 1 Department of Psychiatry and Psychotherapy, University of Regensburg, Universitätsstraße 84, 93053 Regensburg, Germany; 2 Institute for Psychology, University of Regensburg, Universitätsstraße 31, 93053 Regensburg, Germany.

## Abstract

Recent research indicates that tinnitus is related to alterations of neural networks including temporal, parietal, and prefrontal brain regions. The current study examines a rTMS protocol which targets three central nodes of these networks in a two-arm randomized parallel group trial. Overall, 49 patients with chronic tinnitus were randomized to receive either triple-site stimulation (left dorsolateral prefrontal stimulation, 1000 pulses, 20 Hz plus left and right temporoparietal stimulation, 1000 pulses each, 1 Hz) or single-site stimulation (left temporoparietal stimulation, 3000 pulses, 1 Hz). Both groups were treated in ten sessions. Tinnitus severity as measured by the tinnitus questionnaire was assessed before rTMS (day1), after rTMS (day12) and at two follow-up visits (day 90 and day 180). The triple-site protocol was well tolerated. There was a significant reduction in tinnitus severity for both treatment groups. The triple-site group tended to show a more pronounced treatment effect at day 90. However, the measurement time point x group interaction effect was not significant. The current results confirm former studies that indicated a significant reduction of tinnitus severity after rTMS treatment. No significant superiority of the multisite protocol was observed. Future approaches for the enhancement of treatment effects are discussed.

Chronic subjective tinnitus is defined as the perception of sound or noise without presence of a corresponding internal or external sound source. It is a highly prevalent^1^ and for many patients very stressful condition which impairs their everyday lives and mental well-being^2^. There is no cure for tinnitus yet and the development of effective causally oriented treatment options is highly dependent on a more detailed understanding of tinnitus pathophysiology. Traditionally, tinnitus research focused on the peripheral and central auditory system^3^ but in the past years, it has shifted to a more global perspective also considering non-auditory cortical areas^4^,^5^,^6^. It has been shown that tinnitus is accompanied by alterations of functional connectivity within and between several neural networks including temporal, parietal and frontal cortices^7^,^8^,^9^. It is supposed that the tinnitus reaches awareness only if there is a co-activation between the auditory cortex and a “perception network” including parietal and frontal cortices^6^. Correlations of neural processes with clinical tinnitus data suggest that different aspects of the tinnitus percept are encoded by separable networks indeed. For instance, tinnitus loudness was shown to correlate with gamma activity in the auditory cortex^10^ while tinnitus distress has been linked to a general distress network including the anterior cingulate cortex, the dorsolateral prefrontal cortex, the insula and posterior cingulate cortex^11^,^12^. It has also been shown that those networks seem to be functionally interconnected in highly distressed patients^11^. The current study seizes the idea of this network perspective with the aim of improving rTMS treatment for chronic subjective tinnitus.

Having the auditory pathway in mind, many clinical studies have examined the effects of unilateral low-frequency (LF) rTMS of the auditory cortex as a treatment for chronic tinnitus for a review, see Lefaucheur *et al.*^13^. The results of those studies are mixed and the effect size is small emphasizing the need for more effective treatment protocols. One possibility to enhance treatment effects is to increase the number of stimulated areas. In the last years, this optimization strategy has also been increasingly investigated for other neurological or psychiatric indications for rTMS treatment like Major Depression or Parkinson’s disease. Whereas this approach revealed mixed results in depression treatment^14^ the bilateral stimulation of the primary motor cortex has been shown to be superior to unilateral stimulation for the treatment of Parkinson’s disease^13^. The targeted extension of the stimulated areas might therefore represent a promising approach for future rTMS research and might also be useful for the treatment of chronic tinnitus.

The combined stimulation of left temporal and left frontal cortices has already been tested in tinnitus patients. Indeed, patients receiving the combined stimulation protocol showed better long-term symptom improvement than patients who had been treated with single-site temporal stimulation^15^. Another study indicated that the combined protocol appeared in trend to be superior, but the difference was not statistically significant^16^. Based on these results and on the knowledge about the altered functional connectivity between different networks in the tinnitus brain, a new, “triple-site” protocol was recently tested in a pilot, single-arm study^17^. This protocol added another target to the combined protocol of Kleinjung *et al.*^15^ resulting in three stimulation sites: bilateral LF rTMS of the temporoparietal cortex plus HF rTMS of the DLPFC. The triple-protocol targets the most important hubs of the tinnitus network as defined by Schlee *et al.*^7^. In the pilot study, this protocol showed better long-term effects than a historical control group which was treated with unilateral temporal stimulation and - on a descriptive level - also better long-term effects than a historical control group treated with combined left temporal plus left DLPFC stimulation^17^. The current study intends to replicate the results of the pilot study in a randomized controlled trial. We determined whether the stimulation of multiple hubs of the neural networks involved in tinnitus is superior to the standard single-site stimulation protocol.

## Materials and Methods

The presented data come from a two-arm randomized, double-blind parallel-group trial whose design and methods were published in detail in Lehner *et al.*^18^,^19^. The study was registered at Clinical Trials on July 23, 2012 (NCT01663324) and has been approved by the ethics committee of the University of Regensburg (10-101-0169). The study was done in accordance with the approved guidelines. All data were collected at the Department of Psychiatry and Psychotherapy, University of Regensburg between July 2012 and January 2015 (last follow-up visit).

### Subjects

The study was designed to find an interaction effect between group (single-site vs. triple-site) and time (day1, day 12). Based on our pilot data^17^ a small effect size of *f* = 0.1 for this interaction effect was assumed. Although small, such an effect is still an important step in tinnitus management. If the study sample size is determined to provide sufficient power (0.8) for detection of such an effect in a repeated-measures analysis of variance (with α = 0.05), a total of 42 tinnitus patients have to be examined. Due to the complex and time-consuming study design, a higher patient dropout rate than usual was assumed. A total of 50 patients (25 per group) aged between 18 and 70 years were therefore enrolled in the study (see Table 1).

One patient dropped out of the single-site stimulation group after two rTMS sessions due to an increase in tinnitus loudness. Due to this drop-out, data of 49 patients (35 male, 14 female, age 47.11 ± 12.13 years) are reported. All patients suffered from chronic subjective tinnitus with at least moderate handicap as measured with the Tinnitus Handicap Inventory, (THI)^20^ (score ≥ 38). Tinnitus was present in all patients for at least six months. Study exclusion criteria were prior treatment with rTMS, clinical relevant unstable psychiatric, somatic or neurologic comorbidity and all standard exclusion criteria for rTMS treatment. Patients were recruited during routine clinical tinnitus consultations and via announcements in print-media and on the homepage of the tinnitus clinic at the Regensburg University. All patients gave written informed consent.

### Questionnaires and outcome measures

For the assessment of demographical and clinical characteristics patients completed the Tinnitus Sample Case History Questionnaire^21^. All questionnaires listed below were administered on the first treatment day (“day 1”), last treatment day (“day 12”) and during two follow-up visits (“day 90” and “day 180”). Tinnitus severity was assessed using the THI, the Tinnitus Questionnaire (TQ)^22^ and numeric rating scales for tinnitus loudness and annoyance (ranging from 0 = not at all loud/annoying to 10 = extremely loud/annoying). Furthermore, quality of life was measured using the WHO-QoL (World Health Organization Quality of Life) assessment. Depressive symptoms and hyperacusis were assessed using the Major Depression Inventory (MDI) and a German hyperacusis questionnaire (Geräuschüberempfindlichkeitsfragebogen, “GÜF”)^23^. On day 1 and day 12, the hearing level [dB HL] was measured using pure-tone audiometry. It is reported as an average of all thresholds measured bilaterally ranging from 125 Hz to 8 kHz. The comparison between pre and post treatment hearing level served as safety parameter. The primary outcome parameters were defined as a) the change of tinnitus severity from day 1 to day 12 as measured by the TQ score and b) as the number of treatment responders (as defined by a reduction of at least five points in the TQ score). The change in the remaining questionnaires over the four measurement time points (THI, MDI, GÜF, WHO-QoL), the rating scales and the treatment responders on day 90 and day 180 served as secondary outcome parameters.

### rTMS treatment

On the first treatment day, patients were randomized by random group allocation (http://www.random.org) to receive either single site or triple-site rTMS treatment. All patients received ten treatment sessions on ten consecutive working days. Non-blinded study staff assigned patients to the interventions and applied treatment. These persons were not involved in patient management, assessment or data analysis. The triple-site rTMS protocol consisted of HF stimulation of the left dorsolateral prefrontal cortex (DLPFC, 20 Hz, 20 trains, 25 s inter-train interval, 1000 pulses/day) followed by left temporoparietal and right temporoparietal stimulation (1 Hz, 1000 pulses/day each). The three sites were stimulated successively and always in the same order: DLPFC first, then left temporoparietal cortex and right temporoparietal cortex at the end. The single-site group was treated with 3000 pulses/day of the left temporoparietal cortex. Low-frequency rTMS of the left temporoparietal cortex has been the standard approach for rTMS tinnitus treatment during the past years^13^. Both treatment groups received 3000 pulses per session at an intensity of 110% of the resting motor threshold, but–for safety reasons - never higher than 60% of the maximal stimulator output. The resting motor threshold was measured before the first treatment sessions and was defined as the minimal intensity at which at least five of ten motor evoked potentials were 50 μV in amplitude in the right abductor digiti minimi. Treatment was performed with a Medtronic MagPro Option stimulator (Medtronic, Minneapolis, MN, USA) and a 70 mm figure-of-eight coil. The temporoparietal cortices were targeted using the 10–20 system by placing the coil between the temporal (T3/T4) and the parietal (P3/P4) electrode sites^24^,^25^. For targeting the DLPFC, the coil was centered 6 cm anterior from the site over the motor cortex that had been used for defining the resting motor threshold.

### Placebo control group

As the goal of the study was to test superiority of the triple-site stimulation over the standard approach (temporoparietal stimulation), an active stimulation protocol was chosen as control protocol instead of a placebo stimulation, as proposed by recent reviews^13^,^26^. In order to additionally offer a descriptive comparison to placebo stimulation, data of a placebo control group from a previous rTMS study^16^ is presented. Those patients were treated with a sham-coil system (90 mm outer diameter; coil MC-B70, Medtronic, Minneapolis, MN) on ten consecutive working days. The coil was localized at the auditory cortex by using a positron emission tomography-guided neuronavigational system. From the 44 available placebo-datasets^16^, 25 were chosen in order to create a group which matched the triple- and single-site groups with respect to the baseline TQ score, age, gender, tinnitus laterality and tinnitus duration (see Table 1). With respect to outcome measures, only the TQ at day 1, day 12 and day 90 was available. A follow-up period of 180 days is not common in previous published trials and is thus unique for this study. As data of this group were collected earlier by different study staff and under different circumstances, they will not be submitted to statistical analyses. They are meant to provide a qualitative reference point for the possible effects of sham stimulation.

### Statistical analysis

For statistical analyses IBM SPSS Statistics for Windows (Version 22.0, Armonk, NY: IBM Corp.) was applied. Missing values were replaced by using a last observation carried forward (LOCF) procedure, if at least one measurement after rTMS was available. Patients without post-rTMS measurements were not included in the analysis (drop-outs). Concerning the missing values, data of four patients had to be replaced using LOCF on day 90 and data of two patients had to be replaced on day 180. As some of the questionnaires were not filled in correctly, there were some additional missing values for specific questionnaires: data for two patients were missing on day 12 for the MDI and the GUEF questionnaires and data of one additional patient was missing on day 90 for the THI. On day 180, data of two additional patients were missing for the TQ and data for one patient were missing for the rating scales (loudness and annoyance) and for the MDI. In order to test whether the LOCF procedure had an effect on our results, all statistical tests were done twice: for the whole dataset with LOCF and for the smaller subset of data without LOCF. All statistical tests yielded the same results when conducted without LOCF replacement of missing values.

The change of the TQ score from day 1 to day 12 (primary outcome) was tested using an analysis of variance (ANOVA) with the within-subjects factor measurement time point (day 1, day12) and the between subjects factor group. To test for changes in tinnitus severity over all four measurement time points an ANOVA with the within-subjects factor measurement time point (day 1, day 12, day 90, day 180) and between-subjects factor group (single-site vs. triple-site stimulation) was calculated for all questionnaires. The prerequisites for use of ANOVAs were checked for all dependent variables: the homogeneity of variances was tested with Levene’s Test. The result was nonsignificant for all variables except for the MDI on day 12. The F_max_-Test revealed, that an adaptation of the level of significance was not necessary (F_max_ = 2.04). The sphericity of data was checked with Mauchley Tests. In case of significant Mauchley-Tests, Greenhouse-Geisser corrections were applied.

Number of treatment responders on day 12 (primary outcome), day 90 and day 180 were compared using Chi-square tests. Treatment responders were defined as patients with a reduction in the TQ score of at least 5 points^27^. For safety reasons, we compared the hearing level of all patients from pre to post treatment using a paired t-test with the within subjects factor time (day 1, day 12).

## Results

### Adverse Events

Both the left temporoparietal and the triple-site stimulation protocol were well tolerated by the patients. No serious adverse effects were observed. There was no significant change of the hearing level from pre to post rTMS treatment (*t*(48) = −1.38, p = 0.174). The adverse events for both treatment groups are listed in Table 2.

### Statistical analysis

Concerning the primary outcome (change in the TQ score from day 1 to day 12), the effect of measurement time point was significant (F(1,47) = 23.97, p < .001) with the TQ score decreasing from 45.00 (±15.10) to 40.41 (±15.61). The effect of group was not significant (F(1,47) = 0.06; p = 0.802) and there was no significant interaction effect between measurement time point and group for the change in the TQ score from day 1 to day 12 (F(1,47) = 0.003, p = 0.958). Furthermore, there was no significant difference between groups in the responder rates on day 12 (10 responders in each group; χ*2*(1, N = 49) = 0.01, p = 0.906).

**Figure 1:**
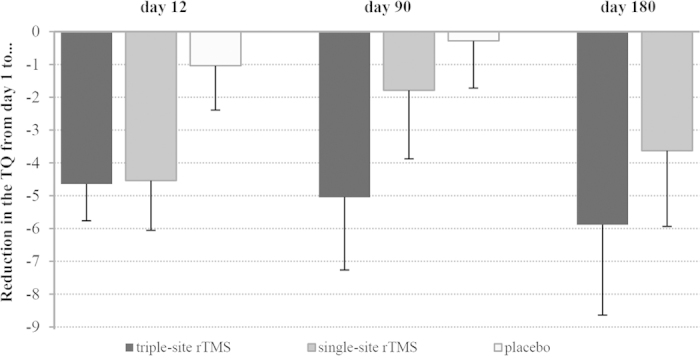
Reduction in the TQ sum score from day 1 to all subsequent measurement time points (for the placebo group only data for day 12 and day 90 were available).

Concerning the secondary outcome measures (ANOVAs comparing all four measurement time points for all questionnaires) significant effects of measurement time point were observed for the TQ (see Fig. 1), the THI and the rating scale “annoyance” (see Table 3). The measurement time point effect for the rating scale “loudness” was marginally significant. For the TQ, post-hoc t-tests revealed significant differences from day 1 to day 12 (*t*(48) = 4.94, *p* > 0.001), from day 1 to day 90 (*t*(48) = 2.26; *p* = 0.029) and to day 180 (*t*(4) = 2.67, *p* = 0.010). The same differences were significant for the THI (day 1 to day 12: *t*(48) = 3.13, *p* = 0.003; day 1 to day 90: *t*(48) = 3.00, *p* = 0.004; day 1 to day 180: *t*(48) = 2.89, *p* = 0.006) and the rating scale “annoyance” (day 1 to day 12: *t*(48) = 2.11, *p* = 0.040; to day 90 *t*(48) = 2.40, *p* = 0.20; to day 180: *t*(48) = 2.31, *p* = 0.025). No significant effects of group were observed (see Table 3). The interaction effects measurement time point x group were not significant either. There was no significant difference between groups in the responder rates on day 90 (9 responders in the single-site group, 13 responders in the triple-site group; χ*2*(1, N = 49) = 1.04, p = 0.308) or on day 180 (10 responders in the single-site group, 14 responders in the triple-site group; χ*2*(1, N = 49) = 1.01, p = 0.316).

### Descriptive comparison with the placebo control group

In Fig. 1, the change of the TQ score from day 1 to all subsequent measurement time points is shown for all three groups separately with negative values indicating a reduction of tinnitus severity. For the placebo group, only data for day 12 and day 90 were available. On a descriptive level, both study groups show more reduction of the TQ score than the placebo group on day 12. On day 90, the triple-site group shows the most pronounced reduction of the TQ, followed by the single-site group. Please note that the group x measurement time point interaction effect was not significant for the two treatment groups. For the placebo group nearly no change of the TQ score was visible on day 90.

## Discussion

Recent studies have suggested that alterations of the connectivity between and within widespread neural networks including frontal, parietal and temporal areas are associated with chronic tinnitus^6^,^7^,^11^,^28^. The current study aimed to use this knowledge about tinnitus pathophysiology for a new treatment option by stimulating three central hubs of these neural networks involved in tinnitus. Results indicate that both the single-site and the triple-site protocols led to a significant reduction of tinnitus severity which emphasizes the potential of rTMS for the treatment of tinnitus. However, the superiority of the triple-site protocol was modest at best (Fig. 1) and the effect sizes were small (Table 3). At first glance these results do not agree with an earlier study from our group^17^. On a descriptive level however, the present results resemble those of the pilot study and a superiority of the triple-site stimulation can be observed 90 days (see Fig. 1) and in trend 180 days after rTMS. The single-site group reported a reduction in tinnitus severity on day 90. This matches exactly what was observed in the pilot study. One possible reason for the lack of statistical significance of the current results in comparison to the pilot data might be that data of the pilot study were not matched with respect to the number of applied pulses. In the pilot study the triple-site group received 4000 pulses per session, the single-site group received only 2000 rTMS pulses per session. There is evidence that treatment with more pulses results in a more pronounced effect both for the treatment of depression^29^ and the treatment of tinnitus^30^. Therefore, the higher dose of the triple-site stimulation might have contributed to its superiority in the pilot study. As the number of pulses was kept constant in the current study design, this lacking dose-effect might be one reason for the unexpected non-significant outcome. This makes clear that future studies investigating multisite stimulation should take the number of pulses into account. If multi-site stimulation involves a higher number of pulses, a possible superiority of multi-site stimulation could be simply the consequence of a higher dose.

Moreover the relative small sample sizes of our study for detecting a differential effect of two active protocols has to be considered in the interpretation of data. The observed effect size of Eta^2^ = 0.013 for the interaction effect between measurement time point and group concerning the TQ is small but might still be in a range of clinical relevance. Although tiny, this effect suggests that there might be some advantage of multisite protocols to evoke a more sustained reduction of tinnitus severity.

The tendency towards a better, albeit modest, long-term effect of the triple-site protocol, which was observed in the current study, is in line with other studies that administered combined treatment protocols^15^,^31^ and indicates the potential of the concept to stimulate multiple sites of a pathologically altered brain network. The idea of stimulating several hubs of the neural networks involved in tinnitus can and should encourage new concepts of multisite-treatment protocols. There are diverse variables which can be varied in future protocols: how many areas should be stimulated in which frequency and in which order? We chose to stimulate all patients in the triple-site group with the same stimulation sequence (first DLPFC, then left and right temporoparietal cortex) in order to stick to the protocol of the pilot study^17^ and in order to be able to use a sample size small enough to enable us to also include EEG and fMRI measurements^18^. Future studies could randomize the order of the stimulated sites in order do find out which sequence of stimulated areas might be most effective. Moreover, it might be more effective if stimulation sites were not treated successively but simultaneously or with a particular timing between the magnetic pulses over different stimulation sites. More knowledge about tinnitus pathophysiology is needed to define treatment protocols which are able to effectively interfere with the tinnitus-specific alterations. Recent studies already provide important information for potential future treatment protocols by presenting increasingly refined knowledge about the neural networks involved in the tinnitus percept. While the current study was motivated by the finding of frequency-specific changes of functional connectivity between temporal, parietal, frontal and cingulate cortices in tinnitus patients^7^, more recent studies define separate distress and loudness networks with e.g. increased electroencephalographic alpha activity in prefrontal areas and increased beta activity in the dorsal anterior cingulate cortex^11^. However, the results of such studies are still mixed with respect to the network hubs considered to be important and the frequencies with which alterations of connectivity can be perceived. Combining treatment studies with brain imaging can help to specify in more detail which changes of functional connectivity are correlated with treatment response and should therefore be targeted^32^. Another promising approach to improve (multi-site) rTMS treatment is customizing brain stimulation to each patient. As tinnitus is a heterogeneous condition the information which neural networks are altered in the tinnitus brain in general may be less relevant than the alterations which are present in the individual tinnitus patient at the very moment we intend to apply rTMS treatment. It is well-known that the effect of rTMS is dependent on the status of the brain at the time the stimulus is applied^33^. It might be therefore a promising task for future studies to identify the optimal treatment protocol for each patient and eventually also for each treatment session separately. A further approach to improve rTMS treatment might be related to increases of the dosage of rTMS. This can be done either by increasing the applied pulses per day or the number of treated days. Here, we stimulated with 3000 pulses per day showing remarkable changes in tinnitus distress which were higher in comparison to a recent meta-analysis^34^ and a retrospective analysis of over 500 patients^35^, where a lower number of pulses per day was used^34^. A higher number of treatment sessions is common in the rTMS treatment of patients suffering from major depression. In these patients, rTMS treatment for four to eight weeks^36^,^37^ has been approved by the Food and Drug Administration in the United States and may also represent a promising approach in improving treatment effects in patients with tinnitus.

## Conclusions

We report a tendency towards a modest, sustained long-term effect of the triple-site stimulation protocol in comparison to the single-site protocol. This descriptive advantage shows that innovative treatment protocols carry potential for a more effective treatment of subjective tinnitus. Future work could aspire to apply novel protocols based on emerging knowledge about tinnitus pathophysiology and, above all, about the individual tinnitus brain.

## Additional Information

**How to cite this article**: Lehner, A. *et al.* Triple-site rTMS for the treatment of chronic tinnitus: a randomized controlled trial. *Sci. Rep.* **6**, 22302; doi: 10.1038/srep22302 (2016).

## Figures and Tables

**Table 1:** Demographical data and clinical characteristics for both treatment groups and the placebo control group.

	single-site rTMS (N = 24)	triple-site rTMS (N = 25)	placebo (N = 25)	group comparisons
age (years)	48.89 ± 10.05	45.39 ± 13.83	52.80 ± 13.32	*F*(2,71) = 2.18; *p* = 0.120
gender	17 m, 7 f	18 m, 7 f	18 m, 7 f	*χ*^*2*^(2) =.01; *p* = 0.995
mean hearing threshold[dB HL]	33.79 ± 13.48	27.71 ± 10.46		*t*(47) = 1.77; *p* = 0.083
tinnitus laterality (r/l/l > r/r > l/both/inside head)	2/5/4/4/8/1	5/6/3/5/5/1	3/6/4/3/7/2	*p* =0.979 (Fisher’s Exact Test)
duration (months)	120.14 ± 118.02	103.93 ± 118.78	95.64 ± 85.46	*F*(2,71) = 0.32; *p* = 0.725
**Questionnaire scores on day 1**				
TQ	44.42 ± 16.66	45.56 ± 13.75	45.24 ± 15.90	*F*(2,71) = 0.04; *p* = 0.965
THI	50.17 ± 22.26	47.36 ± 17.94		*t*(47) = 0.49; *p* = 0.629
MDI	6.25 ± 3.97	7.68 ± 5.60		*t*(47) = −1.03; *p* = 0.310
GÜF (N = 47)	15.70 ± 8.40	16.54 ± 9.34		*t*(45) = −0.33; *p* = 0.746
WHO-QoL Domain 1	16.23 ± 2.50	15.31 ± 2.38		*t*(47) = 1.32; *p* = 0.194
WHO-QoL Domain 2	15.29 ± 2.19	14.13 ± 2.56		*t*(47) = 1.70; *p* = 0.096
WHO-QoL Domain 3	16.21 ± 2.41	15.15 ± 2.95		*t*(47) = 1.38; *p* = 0.175
WHO-QoL Domain 4	17.08 ± 1.54	16.45 ± 2.09		*t*(47) = 1.18; *p* = 0.243

Mean hearing threshold (in dB HL): average of all thresholds measured bilaterally ranging from 125 Hz to 8 kHz. Tinnitus laterality is defined in categories: r: right-sided, l: left-sided, l > r: both sides but louder on the left side; r > l: both sides but louder on the right side; both: both sides; inside head: Tinnitus is perceived in the middle of/ inside the head. TQ: Tinnitus Questionnaire; THI: Tinnitus Handicap Inventory; MDI: Major Depression Inventory; GÜF: Geräuschüberempfindlichkeitsfragebogen (German Hyperacusis Questionnaire); WHO-QoL: World Health Organization-Quality of Life.

**Table 2:** Adverse events for both treatment groups.

	single-site rTMS	triple-site rTMS
transient adverse events		
muscular tension	1	-
headache	6	3
blurred vision	1	-
increase in tinnitus loudness	3	-
mood swings	1	-
dizziness	-	1
feeling of heaviness in the legs	-	1
ongoing adverse events		
increase in tinnitus loudness	3*	-
broadening of the frequency range of the tinnitus	-	1

^*^One of those three patients dropped out after two days of treatment.

**Table 3:** Results from repeated measures analyses of variance.

ANOVA									
	main effect: measurement time point			main effect: group			interaction effect: measurement time point x group		
	F (df)	p	Eta^2^	F (df)	p	Eta^2^	F (df)	p	Eta^2^
TQ	F(2.23, 104.96) = 4.94	0.007*	0.094	F(1, 47) = 0.003	0.954	>0.001	F(2.23, 104.96) = 0.66	0.536	0.013
THI	F(2.48, 116.33) =5.02	0.005*	0.095	F(1, 47) = 0.55	0.463	0.012	F(2.48, 116.33) =1.09	0.349	0.021
MDI	F(2.49, 116.83) = 0.92	0.434	0.019	F(1, 47) = 0.46	0.500	0.10	F(2.49, 116.83) = 1.14	0.330	0.023
GÜF	F(2.34, 105.26) = 1.99	0.134	0.041	F(1, 45) = 0.004	0.948	> 0.001	F(2.34, 105.26) = 1.33	0.267	0.027
loudness	F(2.20, 103.58) = 2.38	0.092#	0.048	F(1, 47) = 0.02	0.893	>.001	F(2.20, 103.58) = 0.39	0.697	0.008
annoyance	F(2.50, 117.28) = 3.17	0.035*	0.063	F(1, 47) = 0.52	0.475	0.011	F(2.50, 117.28) = 0.40	0.719	0.008
WHO-QoL domain 1	F(3, 141) = 0.78	0.505	0.016	F(1, 47) = 0.94	0.338	0.020	F(3, 141) = 0.70	0.555	0.014
WHO-QoL domain 2	F(2.48, 116.66) = 0.16	0.890	0.003	F(1, 47) = 1.15	0.290	0.024	F(2.48, 116.66) = 1.78	0.165	0.036
WHO-QoL domain 3	F(2.28, 107.21) = 0.63	0.596	0.013	F(1, 47) = 2.33	0.134	0.047	F(2.28, 107.21) = 0.14	0.893	0.003
WHO-QoL domain 4	F(2.58, 121.37) = 0.65	0.584	0.013	F(1, 47) = 1.24	0.271	0.026	F(2.58, 121.37) = 0.40	0.590	0.012

^*^α ≤ 0.05

^#^α < 0.10
